# Application of Soft-Computing Methods to Evaluate the Compressive Strength of Self-Compacting Concrete

**DOI:** 10.3390/ma15217800

**Published:** 2022-11-04

**Authors:** Muhammad Nasir Amin, Mohammed Najeeb Al-Hashem, Ayaz Ahmad, Kaffayatullah Khan, Waqas Ahmad, Muhammad Ghulam Qadir, Muhammad Imran, Qasem M. S. Al-Ahmad

**Affiliations:** 1 Department of Civil and Environmental Engineering, College of Engineering, King Faisal University, Al-Ahsa 31982, Saudi Arabia; 2 MaREI Centre, Ryan Institute and School of Engineering, College of Science and Engineering, National University of Ireland Galway, H91 TK33 Galway, Ireland; 3 Department of Civil Engineering, COMSATS University Islamabad, Abbottabad 22060, Pakistan; 4 Department of Environmental Sciences, Abbottabad Campus, COMSATS University Islamabad, Abbottabad 22060, Pakistan; 5 School of Civil and Environmental Engineering (SCEE), National University of Sciences & Technology (NUST), Islamabad 44000, Pakistan

**Keywords:** concrete, self-compacting concrete, compressive strength, prediction models, machine learning

## Abstract

This research examined machine learning (ML) techniques for predicting the compressive strength (CS) of self-compacting concrete (SCC). Multilayer perceptron (MLP), bagging regressor (BR), and support vector machine (SVM) were utilized for analysis. A total of 169 data points were retrieved from the various published articles. The data set was based on 11 input parameters, such as cement, limestone, fly ash, ground granulated blast-furnace slag, silica fume, rice husk ash, coarse aggregate, fine aggregate, superplasticizers, water, viscosity modifying admixtures, and one output with compressive strength of SCC. In terms of properly predicting the CS of SCC, the BR technique outperformed both the SVM and MLP models, as determined by the research results. In contrast to SVM and MLP, the coefficient of determination (R^2^) for the BR model was 0.95, whereas for SVM and MLP, the R^2^ was 0.90 and 0.86, respectively. In addition, a k-fold cross-validation approach was adopted to check the accuracy of the employed models. The statistical measures mean absolute percent error, mean absolute error, and root mean square error ensure the validity of the model. Using sensitivity analysis, the influence of input factors on the intended CS of SCC was also explored. This analysis reveals that the highest contributing parameter towards the CS of SCC was cement with 16.2%, while rice husk ash contributed the least with 4.25% among all the input variables.

## 1. Introduction

Self-compacting concrete (SCC), a type of high-performance concrete (HPC) with a superior ability to deform and resistance to segregation, was developed for the first time in Japan in 1986 [1]. SCC has been utilized in Japan for major office buildings as well as innovative types of extruded tunnels combined with steel fibers [2]. The utilization of SCC reduced the construction site noise level and its impact on the environment. SCC is better than regular concrete for many reasons, including (1) eliminating the need for vibration; (2) lowering construction duration and costs of labor; minimizing noise pollution; (4) enhancing the filling volume of highly crowded structural elements; (5) improving the transition zone among the cement paste and reinforcement or aggregate; (6) limiting concrete’s permeability and increasing its durability [3,4]. The introduction of SCC allows for the exploitation of replacement materials, industrial waste, and other secondary resources, such as mineral chemicals, and generates interest in carrying out this process [5,6,7].

In general, the quality of SCC is determined by its compressive strength (CS), which provides a basic indication of concrete because it is linked to the structure of the hardened mixture [8,9]. Typically, the compressive strength of SCC is determined by costly and time-consuming physical trials, therefore the work productivity will be extremely low [10]. On account of its complicated composition, SCC requires a suitable mixed design procedure in order to achieve its desired qualities [11]. For the selected design procedure, the materials used must be balanced with at least one mineral and one or more chemical additives [12]. The difficulty in improving grain size dispersal and packing particles in stronger cohesion for SCC is met by looking for the optimal balance equivalency among the coarse and fine components and the admixtures [13,14,15]. For this reason, technological advancements make it possible to solve engineering challenges at a lesser cost by employing empirical regression, simulation techniques, and machine learning algorithms [16,17,18]. These approaches enable the forecasting of the CS of SCC based on the proportions of different components in the mixture that has been created such as aggregate, cement, superplasticizers, and water [19,20,21].

In recent decades, machine learning (ML) approaches have emerged as an appealing modelling tool appropriate to a broad array of scientific fields, including materials engineering [22,23,24,25,26,27]. These data sets can be used to build an appropriate surrogate model for predetermined model parameters, hence eliminating the need for costly and time-consuming trials [28]. Considering this, a trend has gained a surge in recent years by using ML techniques to anticipate the CS of concrete material [29,30,31,32,33,34,35]. These methods can be utilized for a number of applications, including regression, classification, correlation, and clustering [36,37,38,39,40]. With the advancement of ML approaches, it is consequently uncomplicated to investigate the CS of SCC along with the concrete’s other properties [41,42]. Thus, to investigate the strength properties of SCC. Asteris et al. [43] employed the artificial neural network algorithm from ML techniques. The study was based on the prediction of 28 days CS of SCC in a limited time period. Awoyera et al. [44] investigate the predictive accuracy of ANN and GEP approaches for the strength properties of SCC. It was reported that both ANN and GEP successfully anticipated the required properties of SCC.

The purpose of this research is to investigate and evaluate the prediction capabilities of three distinct machine learning techniques for the CS of superplasticized self-compacting concrete (SCC). This research is groundbreaking in that it makes a prediction about the CS of SCC on the data set that was chosen by employing both ensemble machine learning methods (boosting regressor) and individual machine learning approaches (SVM, MLP). This research involves the descriptive analysis of the variables, the application of Python codes for running the employed models, statistical checks for the model’s legitimacy, a validation approach for validating the models, and sensitivity analysis to check the impact that the variables have on the predictive outcome. This study has the potential to make a significant contribution to the construction industry’s utilization of novel tools and approaches for investigating the various properties of construction materials in a manner that is economical, takes a limited amount of time, and does not require any physical effort in the laboratory.

## 2. Research Significance

This study presents the implementation of individual machine learning algorithms in addition to ensemble machine learning approaches in order to estimate the compressive strength of self-compacting concrete (SCC). In order to execute the necessary models for the purpose of prediction, the anaconda navigator software was programmed with the Python programming language. Twenty bagging sub-models were trained on the data, and then those models were tuned so that they had the maximum R^2^ value. In addition to this, the test data were confirmed by employing k-fold cross-validation in conjunction with R^2^, MAPE, MAE, and RMSE. Moreover, the statistical model performance index was utilized in order to contrast individual models with ensemble models (e.g., MAPE, MAE, and RMSE). Furthermore, a comparative study of the obtained results and with the results of similar published articles has also been carried out in order to have a better understanding of an accurate model for the forecasting of the concrete’s strength. This was carried out in order to have a better understanding of the accurate model towards the forecasting of the concrete’s strength. In addition, the sensitivity analysis was included in the research in order to analyze the contribution level of each input parameter toward the strength prediction of SCC. This was carried out in order to ensure that the study was as accurate as possible.

## 3. Materials and Methods

**Figure 1:**
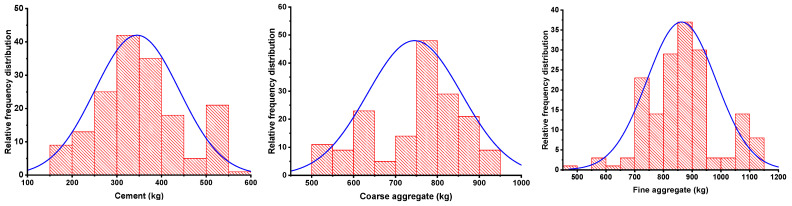

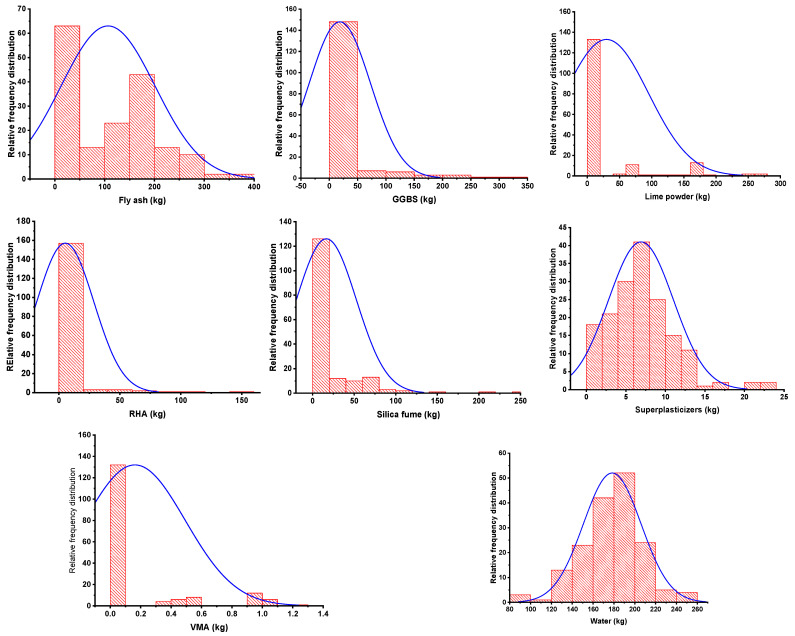
Input parameters’ relative frequency distribution.

**Figure 2:**
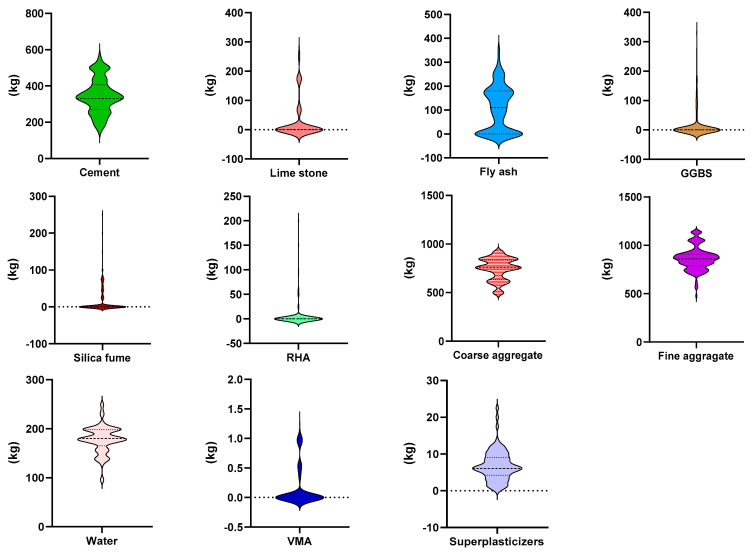
Violin plots distribution of the input parameters.

Python coding (attached in Supplementary Data) in the Anaconda navigator software plays a vital role and was used for running all the employed models. The data set of self-compacting concrete (SCC) used for running the models to anticipate the compressive strength (CS) was retrieved from the literature [45,46,47,48,49,50,51,52,53,54,55,56,57,58,59,60,61,62]. A total of 169 data points (attached in the Supplementary Data) was used for running the selected models. The software automatically splits 70% of the data for training the model and 30% for testing the model. While the k-fold cross validation approach was adopted to validate the required model. To reduce the complexity of the data, the data preprocessing method was adopted. Data preprocessing for data mining addresses one of the most crucial challenges inside the renowned knowledge discovery from data procedure. Data preparation covers data reduction strategies that try to reduce the data’s complexity by recognizing and deleting irrelevant and noisy data items. The model’s analysis was conducted by using the regression and error distribution processes. Eleven input variables, including cement, limestone powder, coarse aggregate, fly ash, water, fine aggregate, GGBS, silica fume, RHA, superplasticizers, and VMA, were introduced for a single outcome such as compressive strength. The selection of these parameters was based on the importance of their effect in the concrete material. The selected input parameters show a significant effect when evaluating their effect using sensitivity analysis. The influence of all the input parameters was also accessed for predicting the CS of SCC through sensitivity analysis. The descriptive statistical analysis was also incorporated for these parameters as listed in Table 1. The validation method has also been adopted to evaluate the precision level of the employed models. Moreover, the histograms give the relative frequency dispersion of all the variables, as shown in Figure 1. A frequency distribution of all the input variables describes how often different values occur in a complete data set. Relative frequency distributions are valuable because they show how common a value is in a data set in comparison to all other values. In addition, violin plot distribution for all the variables is shown in the Figure 2.

## 4. Employed Machine Learning Algorithms

### 4.1. Multilayer Perceptron (MLP)

**Figure 3:**
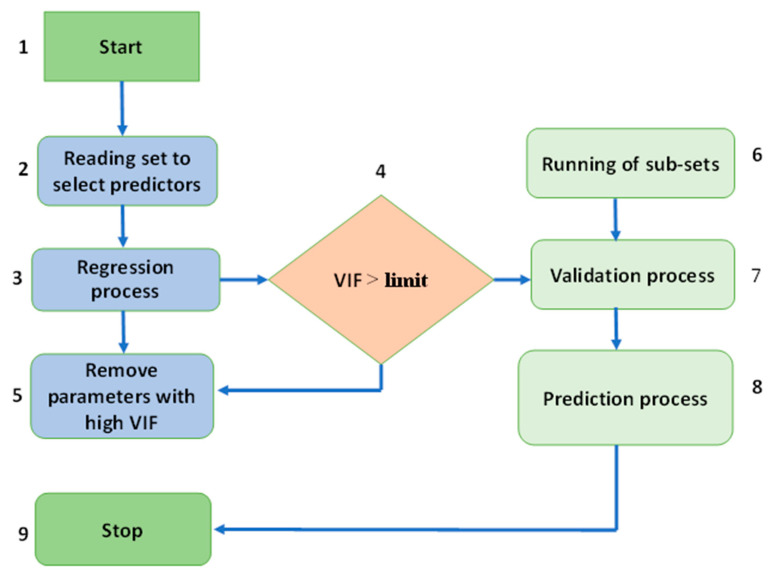
Multilayer perceptron model execution process [63].

An MLP is a type of feedforward ANN that turns a set of inputs into outputs. Between the output and input layers, a targeted graph connects many layers of input nodes. In MLP, the network is trained with backpropagation. It can also connect many loops in a directed graph, with signals moving in only one direction across the nodes. Every entity, with the exception of the input nodes, possesses its very own unique nonlinear activation function. MLPs, which are a form of supervised learning, make use of backpropagation in their learning processes. MLP is often called a deep learning approach because it uses so many layers of neurons. MLP is often used in studies of supervised learning, imputation, parallel distributed processing, and pure science. Machine translation, image recognition, and speech recognition are all examples of applications. To begin, the algorithm selects the predictors that it will be utilized throughout the regression phase in order to locate the variance inflation component (VIF). The VIF then figures out how much an estimated regression coefficient has changed because of collinearity. Figure 3 is a flowchart that shows the whole process of predicting the results of the MLP model.

### 4.2. Support Vector Machine (SVM)

**Figure 4:**
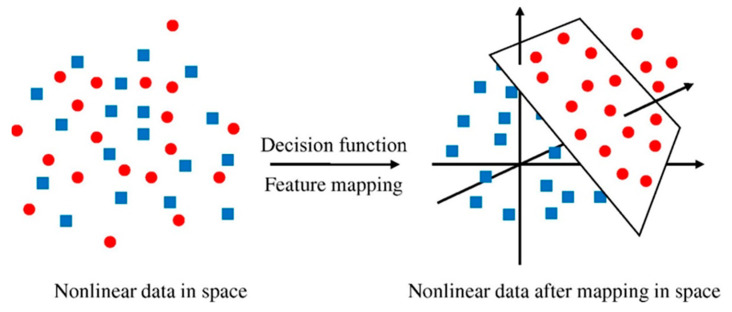
Model mapping of the support vector machine algorithm [64].

**Figure 5:**
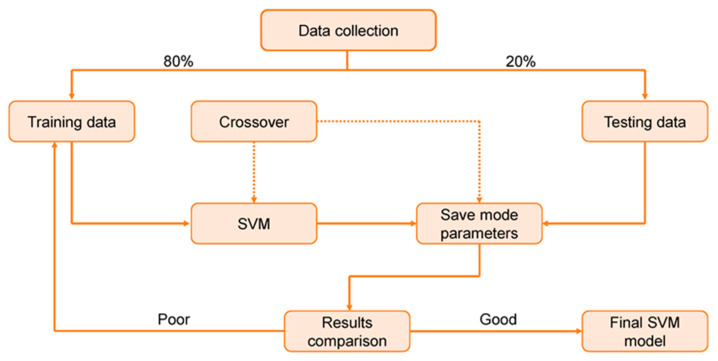
Execution process of the SVM model [65].

SVM refers to a type of algorithm which connected learning algorithms used for evaluating data for both regression and classification. A SVM technique is a description of the samples as points in space that have been drawn in such a way that the patterns of the different classifications are separated by a discrete vector (line/plane) with the largest possible gap. Figure 4 depicts the classification of additional cases based on the side of the vector on which they lie. Figure 5 displays the implementation approach for the SVM model. This model is used to assess the material’s strength, taking into account the influence of multiple factors. The optimization strategy is used to determine the SVM model’s parameters.

### 4.3. Bagging Regressor (BR)

**Figure 6:**
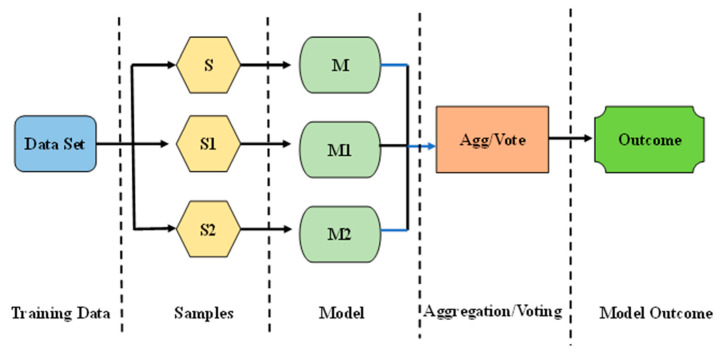
Bagging model execution process for required output.

BR, also referred to as bootstrap aggregation, is a method for combining multiple versions of an anticipated model. Each model is independently trained, then the results are averaged. BR’s primary objective is to attain a lesser divergence than any one model. The process of producing bootstrap samples from a selected data point is known as bootstrapping. The samples are formed by selecting and exchanging data points at random. The characteristics of the resampled data are distinct from those of the original data in its totality. It shows how the data are spread out and tends to keep bootstrapped samples from becoming too similar. This means that the data distribution must stay the same while keeping bootstrapped samples from becoming too similar. This aids in the development of robust models. In addition, bootstrapping helps prevent the overfitting issue. When constructing a model, the utilization of a large number of training data sets results in a decreased likelihood of errors and improved performance when applied to test data. This reduces variation by giving the test set a strong base. Multiple permutations of the model ensure that it is not biased towards an inaccurate outcome. The BR model’s flowchart can be seen in the Figure 6.

## 5. Results and Discussion

### 5.1. MLP Model Outcome

**Figure 7:**
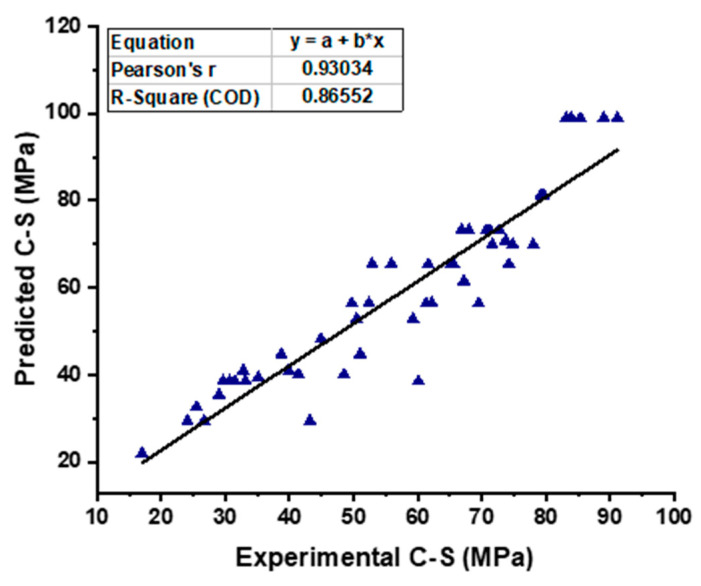
Experimental and predicted outcomes relationship of CS from MLP model.

**Figure 8:**
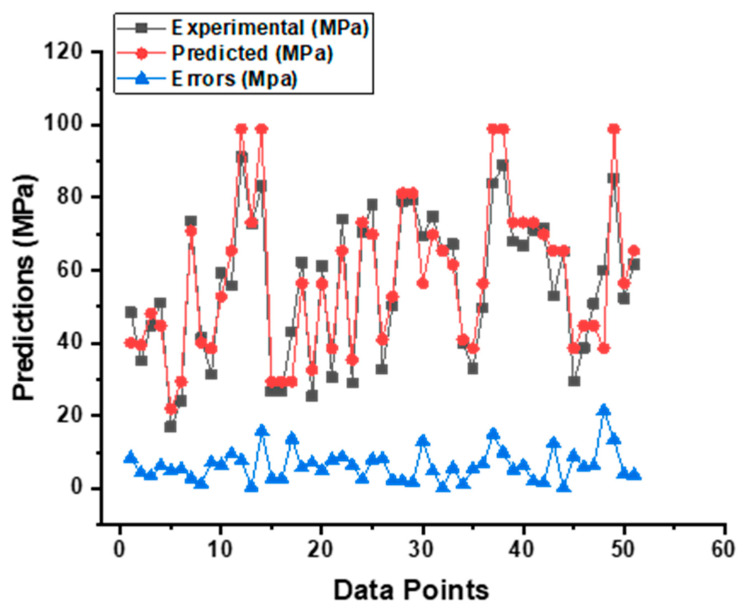
Indication of the error’s difference between the actual and forecasted CS result of SCC from ML model.

Figure 7 shows a depiction of the relationship between the actual and anticipated values for the self-compacting concrete’s (SCC) compressive strength. This relationship gives the coefficient of determination (R^2^) value of 0.86. Figure 8 illustrates the disparity between the actual and expected results. The tabulated information in the figure shows that ‘x’ is the variable that is being explained, and y is the variable that is being investigated. The slope of the line is denoted by the letter b, and ‘a’ is the intercept (the value of y when x is equal to 0). The difference depicts the higher and lower values equal to 21.50 MPa, and 0.18 MPa, respectively. Moreover, it has been noted that the 41.18% of the difference data were found between the minimum value (0.18 MPa) and 5 MPa, and 45.10% of the data were noted among 5 MPa, and 10 MPa. However, only 13.73% of the difference data were located above 10 MPa.

**Figure 9:**
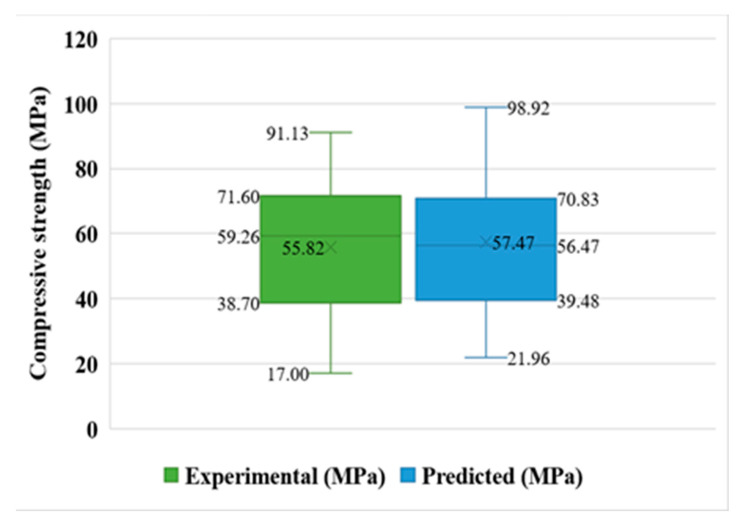
Box plot for predicted and experimental outcomes from MLP model.

The box plot as shown in the Figure 9 gives more statistical information such as the minimum, maximum, median, mean, and first and third quartile values for both the experimental and forecasted outcomes from the test set. The values on the graph clearly indicate the difference of predicted and actual results while comparing.

### 5.2. SVM Model Output

**Figure 10:**
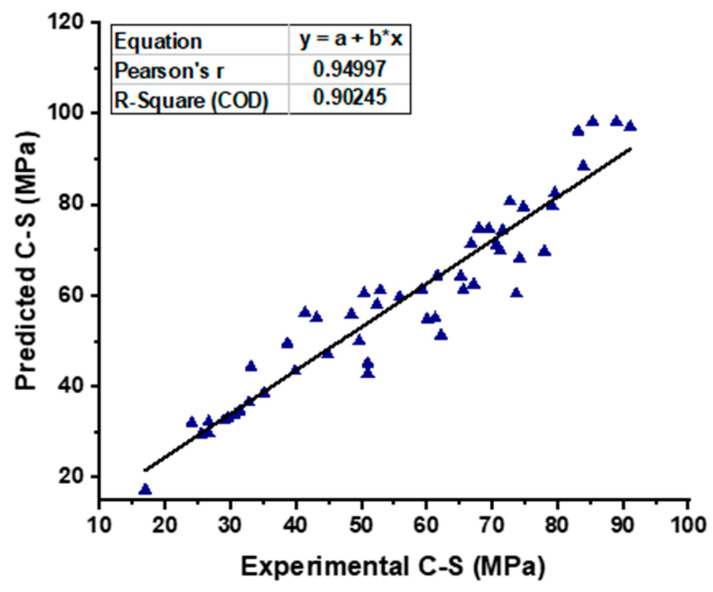
Experimental and predicted outcomes relationship of CS from SVM model.

**Figure 11:**
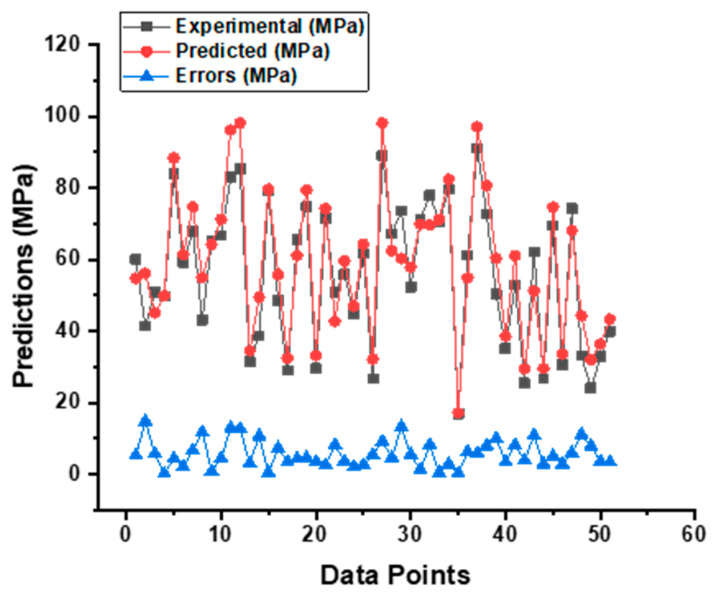
Indication of the error’s difference between the actual and forecasted CS result of SCC from SVM model.

As shown in Figure 10, the SVM model provides a superior link between the experimental CS of SCC and the projected outcome when compared to the MLP model, which results in an R^2^ value of 0.90 having been determined. Figure 11 is an illustration of the distribution of the data, which shows the disparity between the actual and the targeted values. The greatest value, the minimum value, and the average value, all based on this distribution, are 14.81 MPa, 0.21 MPa, and 5.72 MPa, respectively. In addition, 50.98% of these measurements was obtained between 0.21 MPa and 5 MPa, 33.333% of these measurements was obtained between 5 MPa and 10 MPa, and only 15.61% of these measurements was obtained at or above 10 MPa.

**Figure 12:**
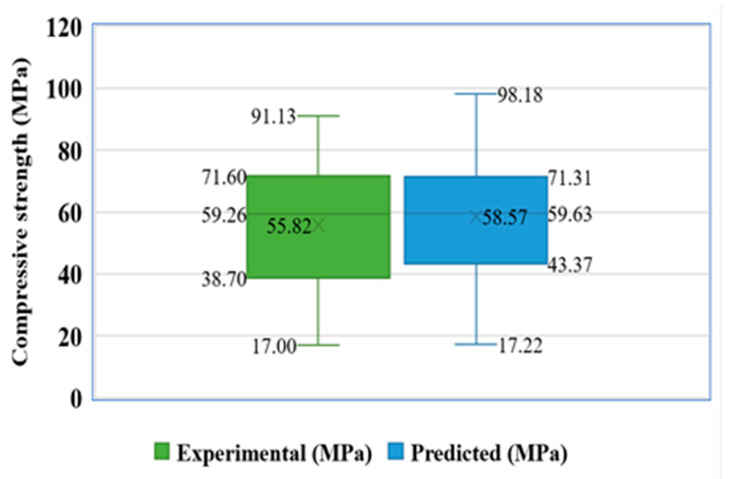
Box plot for predicted and experimental outcomes from SVM model.

In addition, Figure 12 provides additional statistical information, including the minimum, maximum, median, mean, first quartile, and third quartile values for both the experimental and projected outcomes from the test set. The data on the graph make it abundantly evident that there is a disparity between the results that were projected and those that were actually achieved.

### 5.3. BR Model Outcome

**Figure 13:**
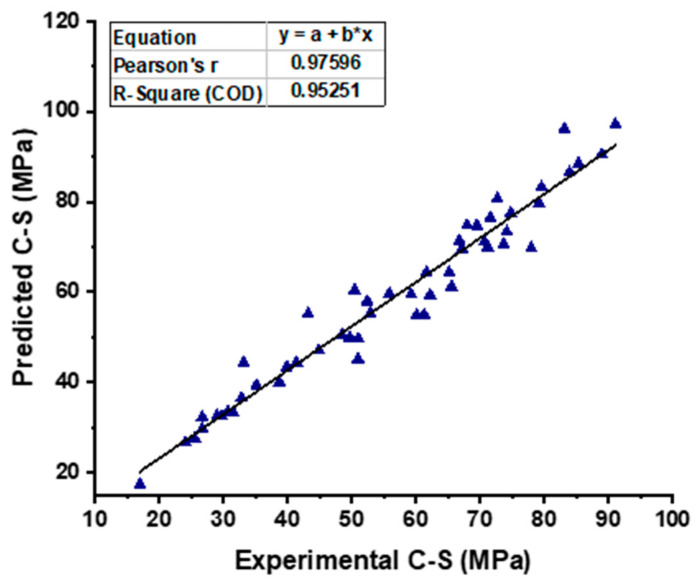
Experimental and predicted outcomes relationship of CS from BR model.

**Figure 14:**
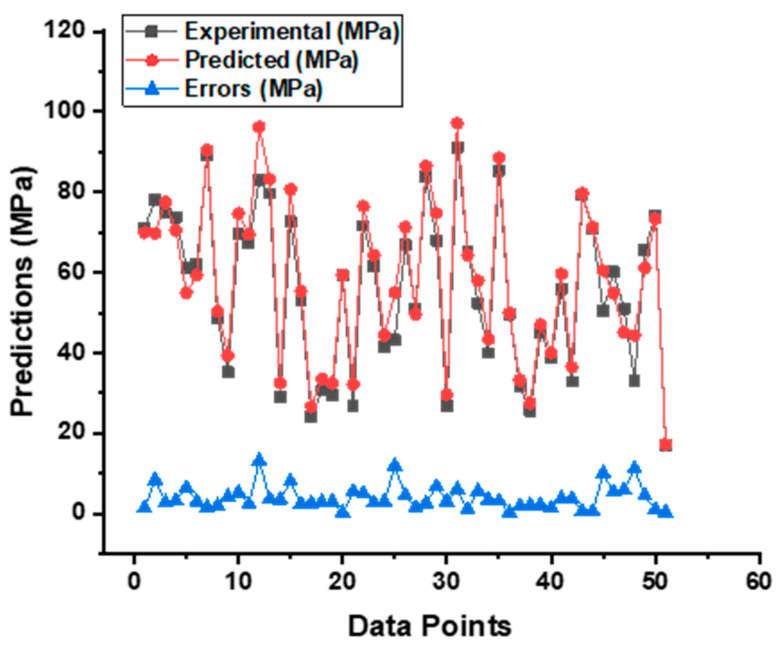
Indication of the error’s difference between the actual and forecasted CS result of SCC from BR model.

As can be seen in Figure 13, the output of the bagging model demonstrates a strong and better relationship with the experimental CS result of the self-compacting concrete than the predictions of the MLP and SVM models, and it gives an R^2^ value of 0.95. This is in contrast to the predictions of the MLP and SVM models. Figure 14 also provides a visual representation of the error’s distribution, which is an additional point of interest. The variation produces data with a maximum of 13.05 MPa, a minimum of 0.16 MPa, and an average of 3.87 MPa, respectively. Additionally, it was seen that 72.55% of this data fell between 0.16 MPa and 5 MPa, while 19.61% of the data were reported to fall between 5 MPa and 10 MPa. However, only 5.88% of these values were found to be higher than the 10 MPa criterion.

**Figure 15:**
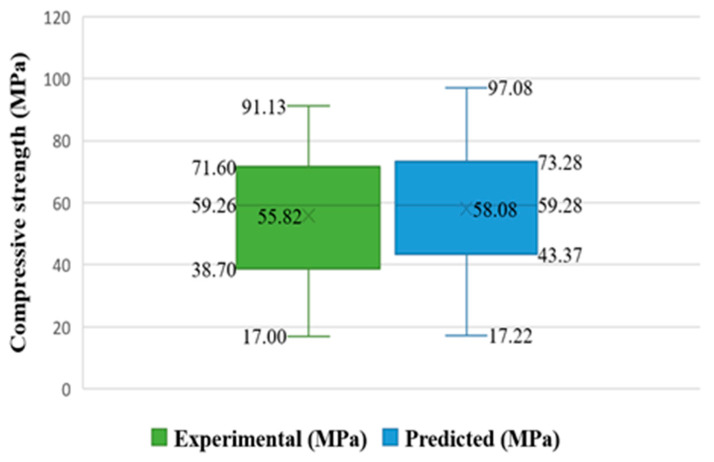
Box plot for predicted and experimental outcomes from BR model.

Moreover, further statistical information is provided in Figure 15, including the minimum, maximum, median, mean, and first and third quartile values for both the experimental and predicted test set results. The discrepancy between the expected and actual outcomes is graphically represented by the graph’s values. The result of the Bagging model seems closer with one another (actual and predicted) as opposed to both SVM and ML models.

### 5.4. K-Fold Cross Validation Outcomes and Statistical Metrics

**Figure 16:**
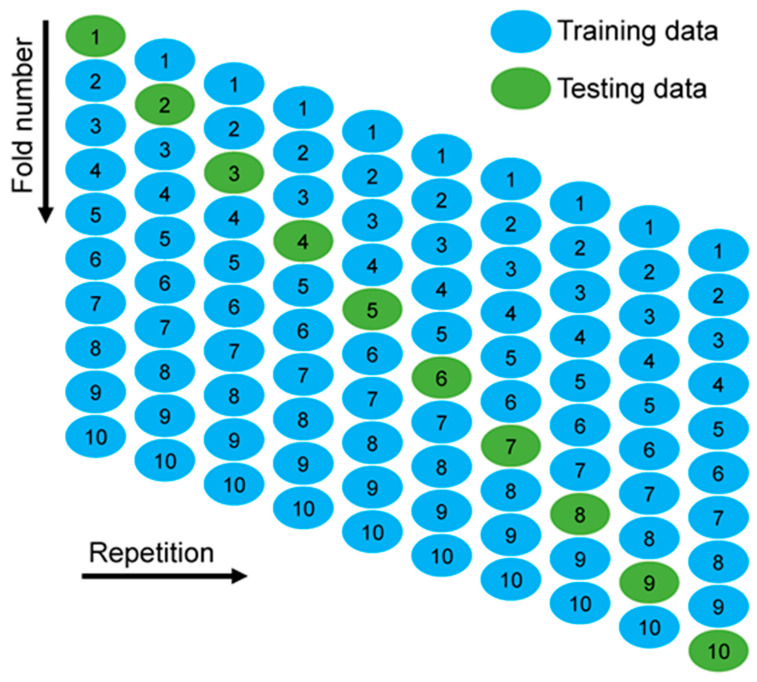
Statistical evaluations of the models used for this investigation [67].

**Figure 17:**
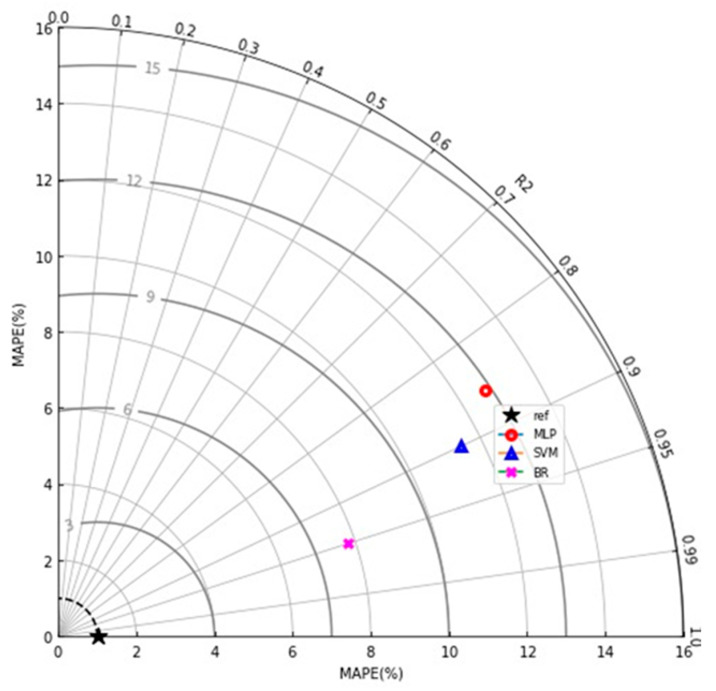
Statistical indication of the model’s performance by Taylor diagram.

K-fold and statistical tests were applied to validate the ML algorithms in use. Typically, the k-fold method is utilized to test the viability of a strategy by arbitrarily distributing and dividing relevant data into 10 groups. As shown in Figure 16, nine groups are used to train machine learning models, while one is used to validate them. The ML approach is more accurate when the errors (MAPE, MAE, and RMSE) are minor and R^2^ is superior. In addition, the technique must be performed 10 times for a desirable outcome. This huge amount of work is a big reason why the model is so accurate. Moreover, the statistical metrics obtained from the models are listed in the Table 2. In the meantime, Figure 17 gives the statistical information about the accuracy level of the employed models for the CS of SCC. This Tylor diagram also indicates the better performance of the bagging model towards the required outcome as compared to SVM and MLP models. The error percent for BR model is less than 8 MPa, while both MLP and SVM models give the same result equal to 12.96 MPa and 11.44 MPa, respectively.

Using Equations (1)–(3) derived from previous research [66], the statistical prediction performance of the techniques was evaluated.

(1) $$\mathrm{MAE}=\frac{1}{n}\overset{n}{\underset{i=1}{\sum }}\left|P_{i}-Ti\right|$$

(2) $$\mathrm{RMSE}=\sqrt{\sum^{\;}\frac{{\left(P_{i}-T_{i}\right)}^{2}}{n}}$$

(3) $$\mathrm{MAPE}=\frac{1}{n}\sum_{t=1}^{n}(A-F/A)$$

where n = number of data points, $P_{i}\;$= anticipated values, and $T_{i}$ = experimental values, *A* is the actual values and *F* is the forecasted values from the data set.

#### Statistical and k-Fold Analysis

**Figure 18:**
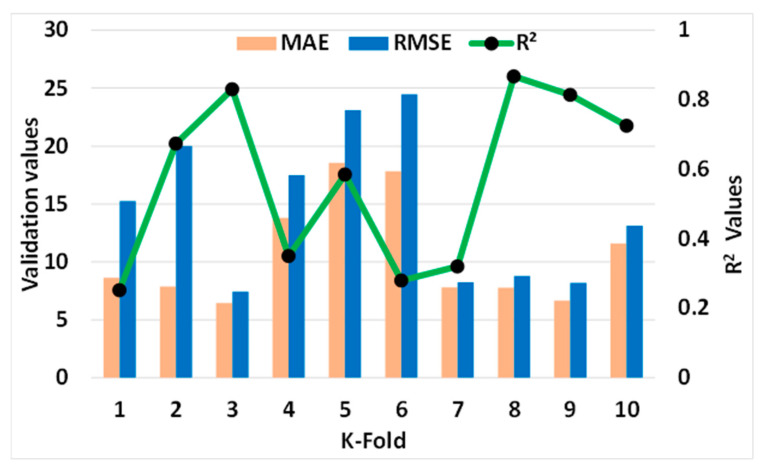
Statistical analysis of MLP model.

**Figure 19:**
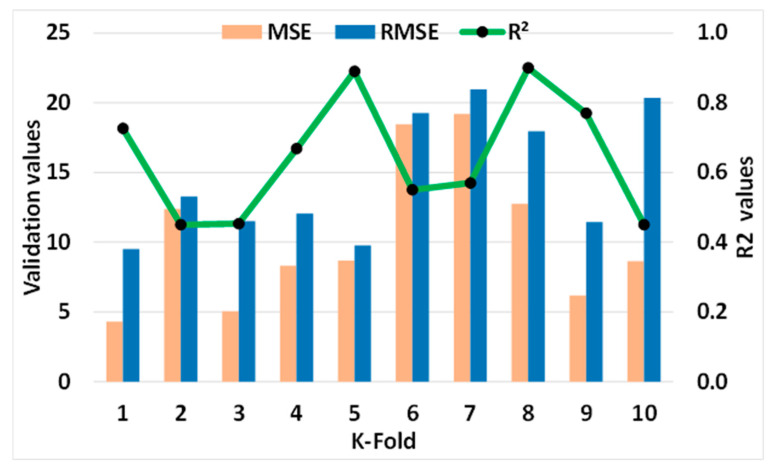
Statistical analysis of SVM model.

**Figure 20:**
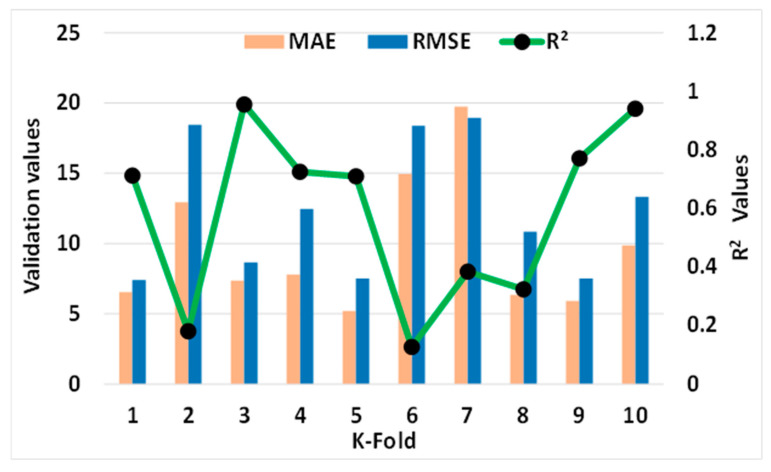
Statistical analysis of BR model.

In order to determine whether or not the model being used is legitimate, a k-fold cross validation check was implemented as a standard. To investigate the results, the statistical metrics were taken into consideration: R^2^, MAE, RMSE. According to the k-fold study, MLP models had higher values of R^2^, MAE, and RMSE, as shown in Figure 18: 0.86, 18.53, and 24.46 MPa, respectively. Similarly, the highest values for the same metrics for SVM models were reported as 0.90, 19.20 MPa, and 20.98 MPa, as shown in Figure 19. However, the higher, lower, and average values of R^2^, MAE, and RMSE for the bagging model were noted as 0.95, 19.74 MPa, and 18.94 MPa, respectively, and can be seen in Figure 20.

### 5.5. Discussion on the Main Findings

**Figure 21:**
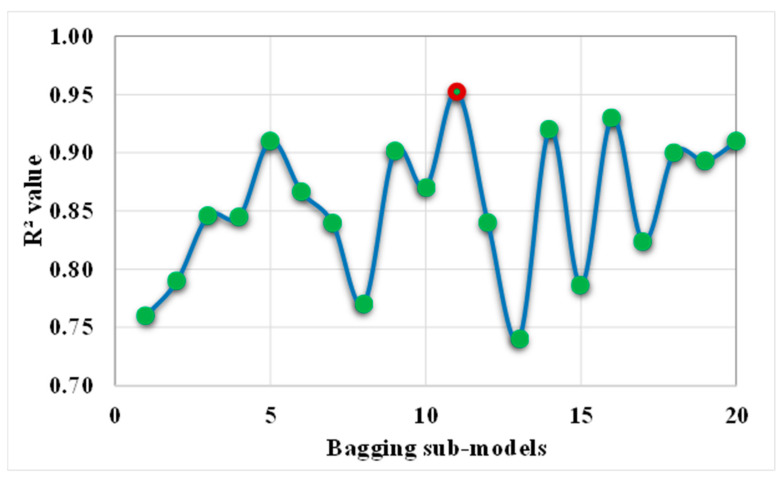
Result of the bagging sub-models.

This study describes the predictive performance of three different types of ML algorithms for the CS of SCC. The multilayer perceptron (MLP), SVM, and bagging regressor (BR) have been investigated for the analysis. Even though MLP and SVM are individual ML techniques, the precision of their predictive results was noted to be within acceptable limits. The BR belongs to the ensemble ML approach, which normally goes through the process of splitting the model into 20-sub models for optimization to have a strong outcome. The result of the bagging sub-models can be seen in the Figure 21. It has been noted that the input parameters and number of data points have a significant effect on the required outcomes. Therefore, the descriptive statistics of the input variables, relative frequency distribution of the input data, and sensitivity analysis for evaluation of their influence on the outcome were incorporated into the study. It was determined that the correlation between the experimental CS result and the prediction CS result from all employed models was satisfactory. The k-fold cross validation approach was also introduced to check the legitimacy of the models. The comparison of the present study with the other relevant studies has also been taken into consideration and found to have a reasonable and better relationship.

### 5.6. Comparison with Other Studies

The result comparison for the application of ML approaches for predicting the same type of outcomes reported in the published articles are listed in the Table 3.

## 6. Sensitivity Analysis

**Figure 22:**
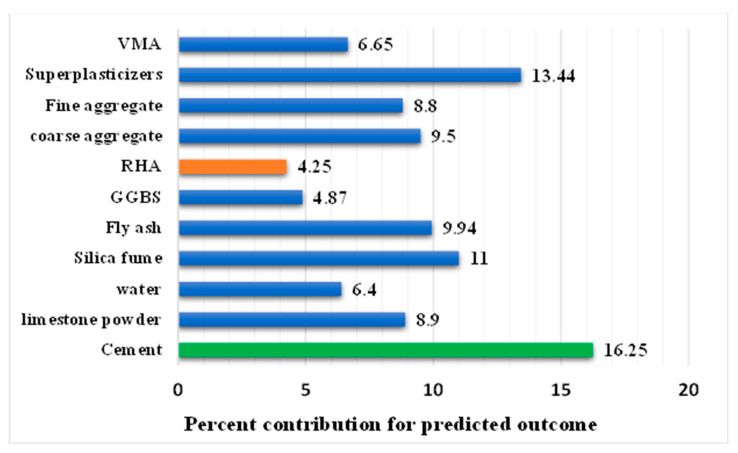
Influence of the input parameters towards the predicted output.

This approach was introduced to investigate the impact of each input variable on the predicted CS of SCC. This analysis reveals that the highest contribution was made by the binding material (cement) by giving 16.25% towards the anticipation of CS of SCC. However, rice husk ash contributed the least (4.25%) to predicting the required outcome. Moreover, the other variables’ impact from the analysis in the descending order were reported for superplasticizers (13.44%), silica fume (11%), fly ash (9.94%), coarse aggregate (9.50%), limestone powder (8.90%), fine aggregate (8.80%), VMA (6.65%), and water (6.40) as depicted in the Figure 22. However, the Equations (4) and (5) were used to calculate the percent contribution of each parameter towards the required outcome.

(4) $$N_{i}=f_{max}\left(x_{i}\right)-f_{min}\left(x_{i}\right)$$

(5) $$S_{i}=\frac{N_{i}}{\sum_{j-i}^{n}N_{j}}$$

where, $f_{max}\left(x_{i}\right)$ and $f_{min}\left(x_{i}\right)$ are the highest and lowest of the anticipated output over the $i^{th}$ output.

## 7. Limitations and Future Perspective

The following are the limitations regarding the application of machine learning approaches along with recommendations for future studies.

- The fact that it is difficult to describe how these algorithms arrive at their findings is a key shortcoming of machine learning.
- A machine learning algorithm is analogous to a black box in that it receives inputs and generates outputs without providing an explanation of how the results were generated.
- In addition to the fact that these algorithms operate in a mysterious manner, machine learning is also susceptible to the fallacy of “garbage in, garbage out”.
- According to this dictum, the output quality is directly proportional to the quality of the data sets used in the analysis.
- The outputs of the algorithm will reflect any mistakes caused by inaccurate labeling of the images/data that are used as inputs.
- Studies based on ML approaches for the prediction of required outcomes can also be enriched with the application of proper hyperparameters for the employed models. Convergence curves based on the RMSE for the selected models and new evaluation indexes such as PI and A-10index can also be included for checking the accuracy level of the employed models. Moreover, the employed models can also be split into training and testing sets to evaluate their performance separately.

## 8. Conclusions

This investigation was predicated on the utilization of supervised ML techniques for the purpose of estimating the CS of SCC. For the purpose of predicting the CS of SCC, the MLP, SVM, and the BR were all investigated. Nevertheless, the following inference can be made based on the findings of the study:

- ML algorithms successfully predict the CS of SCC using python coding.
- In contrast to the SVM and MLP models, the BR model demonstrates outstanding predictive performance with a high degree of precision.
- The high coefficient of determination (R^2^) value (0.95) for the BR model demonstrates its great accuracy in predicting the CS of SCC.
- The K-fold cross validation and statistical metrics also confirm the legitimacy of the employed models.
- The sensitivity analysis provides the impact of each input parameter towards the anticipation of CS of SCC, and it was found that cement contributed the most towards the required outcome.
- This study can also provide civil engineering researchers with a better understanding of how to select the suitable machine learning (ML) algorithms for researching the strength qualities of any form of concrete. The study further provides insight into the significance of input characteristics for the anticipated outcome. Moreover, the experimental approach should also be included for data points (results) to have a better accuracy level of the employed models. Consequently, it is essential to use the required measures and methods for selecting the input variables. Overall, the application of soft computing techniques and tools provides a simple and cost-effective method for analyzing the properties of complicated materials such as concrete.

## Figures and Tables

**Table 1:** Descriptive statistics of variables.

Parameters	Cement (kg/m^3^)	Powder (kg/m^3^)	Fly Ash (kg/m^3^)	GGBS (kg/m^3^)	Silica Fume (kg/m^3^)	RHA (kg/m^3^)	Coarse Aggregate (kg/m^3^)	Fine Aggregate (kg/m^3^)	Water (kg/m^3^)	SP (kg/m^3^)	VMA (kg/m^3^)
Input’s mean	346.04	30.01	106.07	18.75	16.66	5.27	744.66	862.46	178.40	6.90	0.16
Standard Error	7.29	5.04	7.28	4.19	2.78	1.82	8.66	9.30	2.13	0.32	0.03
Input’s median	331.33	0.00	108.75	0.00	0.00	0.00	760.50	860.50	180.03	6.09	0.00
Input’s mode	500.00	0.00	0.00	0.00	0.00	0.00	837.00	900.00	198.00	6.75	0.00
Standard Deviation	94.46	65.35	94.40	54.35	36.08	23.54	112.28	120.54	27.60	4.12	0.33
Range	420.00	272.00	350.00	330.00	250.00	200.00	427.00	657.00	155.50	22.50	1.23
Minimum	150.00	0.00	0.00	0.00	0.00	0.00	500.00	478.00	94.50	0.00	0.00
Maximum	570.00	272.00	350.00	330.00	250.00	200.00	927.00	1135.00	250.00	22.50	1.23
Sum	58,134.24	5041.60	17,818.98	3150.20	2799.00	885.00	125,103.25	144,893.49	29,970.52	1158.45	27.06

**Table 2:** Statistics derived from the employed models.

Algorithms	MAPE (%)	MAE (MPa)	RMSE (MPa)	R^2^
MLP	12.96	6.33	7.71	0.86
SVM	11.44	5.71	6.81	0.90
BR	7.81	3.86	4.86	0.95

**Table 3:** Result comparison with published studies.

Employed Algorithms	R^2^	MAE (MPa)	RMSE (MPa)	References
DT	0.77	6.38	8.952	[18]
GB	0.85	4.95	7.046	
BR	0.91	4.25	5.693	
ANN	0.85	3.42	4.48	[68]
DT	0.86	2.59	3.77	
SVM	0.84	2.93	4.03	
LR	0.85	2.90	4.02	
DT	0.43		3.90	[69]
RF	0.90		1.14	
ANN	0.95		0.91	
DT	0.90	2.62	3.38	[70]
AdaBoost	0.93	2.16	2.62	
BR	0.96	3.75	1.94	
DT	0.82	7.016	10.43	[67]
AdaBoost	0.90	5.19	7.46	
RF	0.90	5.32	7.60	

## Data Availability

The data used in this research have been properly cited and reported in the main text.

## Supplementary Materials

The following supporting information can be downloaded at: https://www.mdpi.com/article/10.3390/ma15217800/s1.

## References

[1] Okamura H., Ouchi M. (2003). Self-compacting concrete. J. Adv. Concr. Technol..

[2] Ouchi M., Nakamura S.-a., Osterberg T., Hallberg S., Lwin M. (2003). Applications of self-compacting concrete in Japan, Europe and the United States. Proceedings of the 5th International Symposium on High Performance Computing (ISHPC).

[3] Shi C., Yang X., Yu Z., Khayat H. Design and application of self-compacting lightweight concretes. Proceedings of the SCC’2005-China: 1st International Symposium on Design, Performance and Use of Self-Consolidating Concrete.

[4] Ozawa K. High-performance concrete based on the durability design of concrete structures. Proceedings of the Second East Asia-Pacific Conference on Structural Engineering and Construction.

[5] Long G., Gao Y., Xie Y., Materials B. (2015). Designing more sustainable and greener self-compacting concrete. Constr. Build. Mater..

[6] Najim K.B., Al-Jumaily I., Atea A.M. (2016). Characterization of sustainable high performance/self-compacting concrete produced using CKD as a cement replacement material. Constr. Build. Mater..

[7] Kumar K.R., Shyamala G., Awoyera P., Vedhasakthi K., Olalusi O. (2021). Cleaner production of self-compacting concrete with selected industrial rejects-an overview. Silicon.

[8] Domone P. (2007). A review of the hardened mechanical properties of self-compacting concrete. Cem. Concr. Compos..

[9] Viacava I.R., de Cea A.A., De Sensale G.R. (2012). Self-compacting concrete of medium characteristic strength. Constr. Build. Mater..

[10] Bradu A., Cazacu N., Florea N., Mihai P. (2016). Compressive strength of self compacting concrete. Bul. Inst. Politeh. Din Lasi. Sect. Constr. Arhit..

[11] Malhotra H. (1956). The effect of temperature on the compressive strength of concrete. Mag. Concr. Res..

[12] Yeh I.-C. (2006). Analysis of strength of concrete using design of experiments and neural networks. J. Mater. Civ. Eng..

[13] Tangadagi R.B., Manjunatha M., Seth D., Preethi S. (2021). Role of mineral admixtures on strength and durability of high strength self compacting concrete: An experimental study. Materialia.

[14] Uysal M., Sumer M. (2011). Performance of self-compacting concrete containing different mineral admixtures. Constr. Build. Mater..

[15] Uysal M., Yilmaz K., Composites C. (2011). Effect of mineral admixtures on properties of self-compacting concrete. Cem. Concr. Compos..

[16] Xu Y., Ahmad W., Ahmad A., Ostrowski K.A., Dudek M., Aslam F., Joyklad P. (2021). Computation of High-Performance Concrete Compressive Strength Using Standalone and Ensembled Machine Learning Techniques. Materials.

[17] Song Y., Zhao J., Ostrowski K.A., Javed M.F., Ahmad A., Khan M.I., Aslam F., Kinasz R. (2022). Prediction of Compressive Strength of Fly-Ash-Based Concrete Using Ensemble and Non-Ensemble Supervised Machine-Learning Approaches. Appl. Sci..

[18] Khan K., Ahmad W., Amin M.N., Aslam F., Ahmad A., Al-Faiad M.A. (2022). Comparison of Prediction Models Based on Machine Learning for the Compressive Strength Estimation of Recycled Aggregate Concrete. Materials.

[19] Ahmad A., Farooq F., Ostrowski K.A., Śliwa-Wieczorek K., Czarnecki S. (2021). Application of Novel Machine Learning Techniques for Predicting the Surface Chloride Concentration in Concrete Containing Waste Material. Materials.

[20] Ahmad A., Ostrowski K.A., Maślak M., Farooq F., Mehmood I., Nafees A. (2021). Comparative Study of Supervised Machine Learning Algorithms for Predicting the Compressive Strength of Concrete at High Temperature. Materials.

[21] Ahmad A., Chaiyasarn K., Farooq F., Ahmad W., Suparp S., Aslam F. (2021). Compressive Strength Prediction via Gene Expression Programming (GEP) and Artificial Neural Network (ANN) for Concrete Containing RCA. Buildings.

[22] Shang M., Li H., Ahmad A., Ahmad W., Ostrowski K.A., Aslam F., Joyklad P., Majka T.M. (2022). Predicting the Mechanical Properties of RCA-Based Concrete Using Supervised Machine Learning Algorithms. Materials.

[23] Ahmad A., Ahmad W., Chaiyasarn K., Ostrowski K.A., Aslam F., Zajdel P., Joyklad P. (2021). Prediction of Geopolymer Concrete Compressive Strength Using Novel Machine Learning Algorithms. Polymers.

[24] Cui L., Yang S., Chen F., Ming Z., Lu N., Qin J. (2018). A survey on application of machine learning for Internet of Things. Int. J. Mach. Learn. Cybern..

[25] Pandey P.K., Aggarwal P., Aggarwal Y., Aggarwal S. (2022). Prediction of Compressive Strength of Self-Compacting Concrete Containing Silica’s Using Soft Computing Techniques. Applications of Computational Intelligence in Concrete Technology.

[26] Onyelowe K.C., Ebid A.M., Riofrio A., Baykara H., Soleymani A., Mahdi H.A., Jahangir H., Ibe K. (2022). Multi-Objective Prediction of the Mechanical Properties and Environmental Impact Appraisals of Self-Healing Concrete for Sustainable Structures. Sustainability.

[27] Andalib A., Aminnejad B., Lork A. (2022). Grey Wolf Optimizer-Based ANNs to Predict the Compressive Strength of Self-Compacting Concrete. Appl. Comput. Intell. Soft Comput..

[28] Wang H., Ma C., Zhou L. A brief review of machine learning and its application. Proceedings of the 2009 International Conference on Information Engineering and Computer Science.

[29] Sonebi M., Cevik A., Grünewald S., Walraven J. (2016). Modelling the fresh properties of self-compacting concrete using support vector machine approach. Constr. Build. Mater..

[30] Aiyer B.G., Kim D., Karingattikkal N., Samui P., Rao P.R. (2014). Prediction of compressive strength of self-compacting concrete using least square support vector machine and relevance vector machine. KSCE J. Civ. Eng..

[31] Asteris P., Kolovos K., Douvika M., Roinos K. (2016). Prediction of self-compacting concrete strength using artificial neural networks. Eur. J. Environ. Civ. Eng..

[32] Dutta S., Murthy A.R., Kim D., Samui P. (2017). Prediction of compressive strength of self-compacting concrete using intelligent computational modeling. Comput. Mater. Contin.

[33] Yuan X., Tian Y., Ahmad W., Ahmad A., Usanova K.I., Mohamed A.M., Khallaf R. (2022). Machine Learning Prediction Models to Evaluate the Strength of Recycled Aggregate Concrete. Materials.

[34] Sarkhani Benemaran R., Esmaeili-Falak M., Javadi A. (2022). Predicting resilient modulus of flexible pavement foundation using extreme gradient boosting based optimised models. Int. J. Pavement Eng..

[35] Wang J., Wu F. (2022). New hybrid support vector regression methods for predicting fresh and hardened properties of self-compacting concrete. J. Intell. Fuzzy Syst..

[36] Kumar B.N., Kumar P.P. (2022). Prediction on Flexural strength of High Strength Hybrid Fiber Self Compacting Concrete by using Artificial Intelligence. J. Artif. Intell..

[37] Nehdi M., El Chabib H., El Naggar M.H. (2001). Predicting performance of self-compacting concrete mixtures using artificial neural networks. Mater. J..

[38] Nguyen T.T., Pham Duy H., Pham Thanh T., Vu H.H. (2020). Compressive Strength Evaluation of Fiber-Reinforced High-Strength Self-Compacting Concrete with Artificial Intelligence. Adv. Civ. Eng..

[39] Asri Y.E., Aicha M.B., Zaher M., Alaoui A.H. (2022). Prediction of compressive strength of self-compacting concrete using four machine learning technics. Mater. Today Proc..

[40] de-Prado-Gil J., Palencia C., Silva-Monteiro N., Martínez-García R. (2022). To predict the compressive strength of self compacting concrete with recycled aggregates utilizing ensemble machine learning models. Case Stud. Constr. Mater..

[41] Balf F.R., Kordkheili H.M., Kordkheili A.M. (2021). A New method for predicting the ingredients of self-compacting concrete (SCC) including fly ash (FA) using data envelopment analysis (DEA). Arab. J. Sci. Eng..

[42] Kovačević M., Lozančić S., Nyarko E.K., Hadzima-Nyarko M. (2021). Modeling of compressive strength of self-compacting rubberized concrete using machine learning. Materials.

[43] Asteris P.G., Kolovos K.G. (2019). Self-compacting concrete strength prediction using surrogate models. Neural Comput. Appl..

[44] Awoyera P.O., Kirgiz M.S., Viloria A., Ovallos-Gazabon D. (2020). Estimating strength properties of geopolymer self-compacting concrete using machine learning techniques. J. Mater. Res. Technol..

[45] Baskar I., Ramanathan P., Venkatasubramani R. (2012). Influence of silica fume on properties of self-compacting concrete. Int. J. Emerg. Trends Eng. Dev..

[46] Brouwers H., Radix H. (2005). Self-compacting concrete: Theoretical and experimental study. Cem. Concr. Res..

[47] Fathi A., Shafiq N., Nuruddin M., Elheber A. (2013). Study the effectiveness of the different pozzolanic material on self-compacting concrete. ARPN J. Eng. Appl. Sci..

[48] Felekoğlu B., Türkel S., Baradan B. (2007). Effect of water/cement ratio on the fresh and hardened properties of self-compacting concrete. Build. Environ..

[49] Gandage A., Ram V., Sivakumar M., Vasan A., Venu M., Yaswanth A. Optimization of class C flyash dosage in self-compacting concrete for pavement applications. Proceedings of the International Conference on Innovations in Concrete for Meeting Infrastructure Challenge.

[50] Gesoğlu M., Güneyisi E., Özbay E. (2009). Properties of self-compacting concretes made with binary, ternary, and quaternary cementitious blends of fly ash, blast furnace slag, and silica fume. Constr. Build. Mater..

[51] Gesoğlu M., Özbay E. (2007). Structures. Effects of mineral admixtures on fresh and hardened properties of self-compacting concretes: Binary, ternary and quaternary systems. Mater. Struct..

[52] Grdić Z., Despotović I., Topličić-Ćurčić G. (2008). Properties of self-compacting concrete with different types of additives. Facta Univ.-Ser. Archit. Civ. Eng..

[53] Memon S.A., Shaikh M.A., Akbar H. (2011). Utilization of Rice Husk Ash as viscosity modifying agent in Self Compacting Concrete. Constr. Build. Mater..

[54] Phani S., Sekhar S., Rao S., Sravana P. (2013). High strength self-compacting concrete using mineral admixtures. Indian Concr J.

[55] Rahman M.E., Muntohar A.S., Pakrashi V., Nagaratnam B., Sujan D. (2014). Self compacting concrete from uncontrolled burning of rice husk and blended fine aggregate. Mater. Des..

[56] Rao N., Rao P., Sravana P., Sekhar T. Studies on relationship of water-powder ratio and compressive strength of self-compacted concrete. Proceedings of the 34th Conference on Our World in Concrete and Structures.

[57] Şahmaran M., Yaman İ.Ö., Tokyay M. (2009). Transport and mechanical properties of self consolidating concrete with high volume fly ash. Cem. Concr. Compos..

[58] Sfikas I.P., Trezos K.G. (2013). Effect of composition variations on bond properties of self-compacting concrete specimens. Constr. Build. Mater..

[59] Siddique R. (2011). Properties of self-compacting concrete containing class F fly ash. Mater. Des..

[60] Sonebi M. (2004). Medium strength self-compacting concrete containing fly ash: Modelling using factorial experimental plans. Cem. Concr. Res..

[61] Sukumar B., Nagamani K., Raghavan R.S., Materials B. (2008). Evaluation of strength at early ages of self-compacting concrete with high volume fly ash. Constr. Build. Mater..

[62] Valcuende M., Marco E., Parra C., Serna P. (2012). Influence of limestone filler and viscosity-modifying admixture on the shrinkage of self-compacting concrete. Cem. Concr. Res..

[63] Khan K., Ahmad A., Amin M.N., Ahmad W., Nazar S., Arab A.M.A. (2022). Comparative Study of Experimental and Modeling of Fly Ash-Based Concrete. Materials.

[64] Ling H., Qian C., Kang W., Liang C., Chen H. (2019). Combination of Support Vector Machine and K-Fold cross validation to predict compressive strength of concrete in marine environment. Constr. Build. Mater..

[65] Amin M.N., Khan K., Ahmad W., Javed M.F., Qureshi H.J., Saleem M.U., Qadir M.G., Faraz M.I. (2022). Compressive Strength Estimation of Geopolymer Composites through Novel Computational Approaches. Polymers.

[66] Nazar S., Yang J., Ahmad A., Shah S.F.A. (2022). Comparative study of evolutionary artificial intelligence approaches to predict the rheological properties of fresh concrete. Mater. Today Commun..

[67] Wang Q., Ahmad W., Ahmad A., Aslam F., Mohamed A., Vatin N.I. (2022). Application of Soft Computing Techniques to Predict the Strength of Geopolymer Composites. Polymers.

[68] Güçlüer K., Özbeyaz A., Göymen S., Günaydın O. (2021). A comparative investigation using machine learning methods for concrete compressive strength estimation. Mater. Today Commun..

[69] Chopra P., Sharma R.K., Kumar M., Chopra T. (2018). Comparison of machine learning techniques for the prediction of compressive strength of concrete. Adv. Civ. Eng..

[70] Ahmad A., Ahmad W., Aslam F., Joyklad P. (2022). Compressive strength prediction of fly ash-based geopolymer concrete via advanced machine learning techniques. Case Stud. Constr. Mater..

